# One Year of the COVID-19 Pandemic in Galicia: A Global View of Age-Group Statistics during Three Waves

**DOI:** 10.3390/ijerph18105104

**Published:** 2021-05-12

**Authors:** Iván Area, Henrique Lorenzo, Pedro J. Marcos, Juan J. Nieto

**Affiliations:** 1 Universidade de Vigo, 32004 Ourense, Spain; area@uvigo.gal; 2 Research Center in Technologies, Energy and Industrial Processes CINTECX, GeoTECH Research Group, Universidade de Vigo, 36310 Vigo, Spain; hlorenzo@uvigo.es; 3 Dirección Asistencial, Complejo Hospitalario Universitario de A Coruña (CHUAC), Instituto de Investigación Biomédica de A Coruña (INIBIC), Universidade da Coruña, Sergas, 15006 A Coruña, Spain; pedro.jorge.marcos.rodriguez@sergas.es; 4 Instituto de Matemáticas, Universidade de Santiago de Compostela, 15782 Santiago de Compostela, Spain

**Keywords:** SARS-CoV-2, new infected, hospital admissions, intensive care unit admission, deceased

## Abstract

In this work we look at the past in order to analyze four key variables after one year of the COVID-19 pandemic in Galicia (NW Spain): new infected, hospital admissions, intensive care unit admissions and deceased. The analysis is presented by age group, comparing at each stage the percentage of the corresponding group with its representation in the society. The time period analyzed covers 1 March 2020 to 1 April 2021, and includes the influence of the B.1.1.7 lineage of COVID-19 which in April 2021 was behind 90% of new cases in Galicia. It is numerically shown how the pandemic affects the age groups 80+, 70+ and 60+, and therefore we give information about how the vaccination process could be scheduled and hints at why the pandemic had different effects in different territories.

## 1. Introduction

Since the beginning of the pandemic of COVID-19, a number of attempts to predict its evolution have been published, helping the health authorities with their strategies. The mathematical tools used vary from compartmental models [1,2,3,4,5], including fractional derivatives [6,7], to statistical tools [8,9,10]. With the aim of helping health authorities to avoid hospital resource collapses, some works have been used, e.g., to predict the numbers of required beds at intensive care units [11].

The first case reported in Galicia was detected on 4th March 2020, and since then three “waves” of the pandemic have happened. That day we ran several compartmental models predicting the peak of the first wave of the COVID-19 epidemic to occur on 5 April 2020, as was the case [1]. Since then, we have improved our models and have been collaborating with different research groups, institutions and authorities.

The main aim of this work was to perform a descriptive statistical analysis [12,13,14] of real data after one year of the pandemic in Galicia (Spain), by considering newly infected individuals, admissions to hospitals, admissions to intensive care units and deceased individuals.

Regarding the target population, Galicia is an autonomous community located in the northwest of Spain, which has a population of about 2,700,000 and a total area of 29,574 km^2^. Furthermore, since the impact of the disease varies a lot due to age, we decided to take an approach based on age groups.

## 2. Methods

Descriptive statistics can be understood as specific methods used to calculate, describe and summarize collected research data in a logical, meaningful and efficient way [15]. Descriptive statistics are reported numerically in the text or in tables, and graphically in figures [16].

The official data considered about COVID-19 come from the institute of health Carlos III (Instituto de Salud Carlos III, ISCIII) of Spain, which has been collecting the data from the different autonomous regions of Spain since the beginning of the pandemic [17]. As for the living population in Galicia, we have considered the official data published by the Galician institute of statistics (Instituto Galego de Estatística, IGE) [18]. According to this official body, the total population of Galicia is 2,698,718 individuals, with the following populations in each age group:

In Table 1 it is noted that population older than 80 is 8.69% of the total, that 47.55% of the total population is older than 50, and that almost 2/3 of the total population is older than 40. On the other hand, only 15.8% of the population is less than 20 years old, and less than 1/4 of the total population is younger than 30. In other words, Galicia is a somewhat elderly society.

Regarding information about pandemic variables, daily data from ISCIII can be accessed in some public websites; in particular, we have used [19]. Information about (i) new infected, (ii) hospital admissions, (iii) intensive care unit admissions and (iv) deceased was collected from that repository, which also organizes data by Spanish provinces, gender and age groups. Data were pre-processed to select the four Galician provinces, discard gender info (which is not the focus of this study) and organize data by nine age groups regarding the four key variables considered.

**Figure 1:**
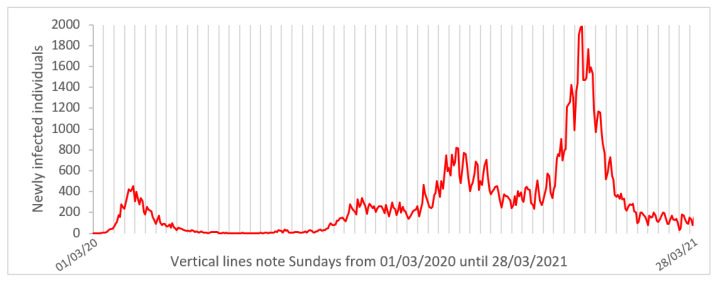
Daily count of newly infected individuals in Galicia since the beginning of the pandemic (raw data). Vertical grid lines separate each week and note Sundays to point out the “weekend effect”: most of them correspond to local minima in daily new infected values.

As we have done in previous works for predicting the evolution of the pandemic [6,11], we have considered for each day the mean of seven previous days’ data. This was done for all variables. There were some reasons for doing this. We wanted to point out that during the first months of the pandemic, many efforts were made to save lives and to accommodate patients, sometimes at the cost of precise counting. Moreover, even in more recent months, we have observed what we refer as “weekend effect”: normally, Sunday’s data about newly infected individuals show a local minimum (Figure 1). Data counting is also affected by local holidays. The idea of considering the mean of 7 previous days tries to compensate for the effect of these artificial local minima, and to avoid considering more than one Sunday in the mean. This also contributes to smoothing the data.

We present the data about the four variables by considering age groups. We considered three time-intervals: The first one corresponds to the so-called first wave, from 4 March 2020 to mid-June 2020. The second corresponds to the full period of pandemic. Finally, we have performed a detailed analysis of 80+ age group’s data evolution, from 1 January 2021 to 1 April 2021, as in the last days of December 2020 vaccination started in this group, and the first B.1.1.7 lineage infection (British strain) was detected in Galicia [20].

## 3. Results

In this section the results are presented in graphs and tables. The comments regarding these results have been organized into four sub-sections, each for a specific variable under study.

**Figure 2:**
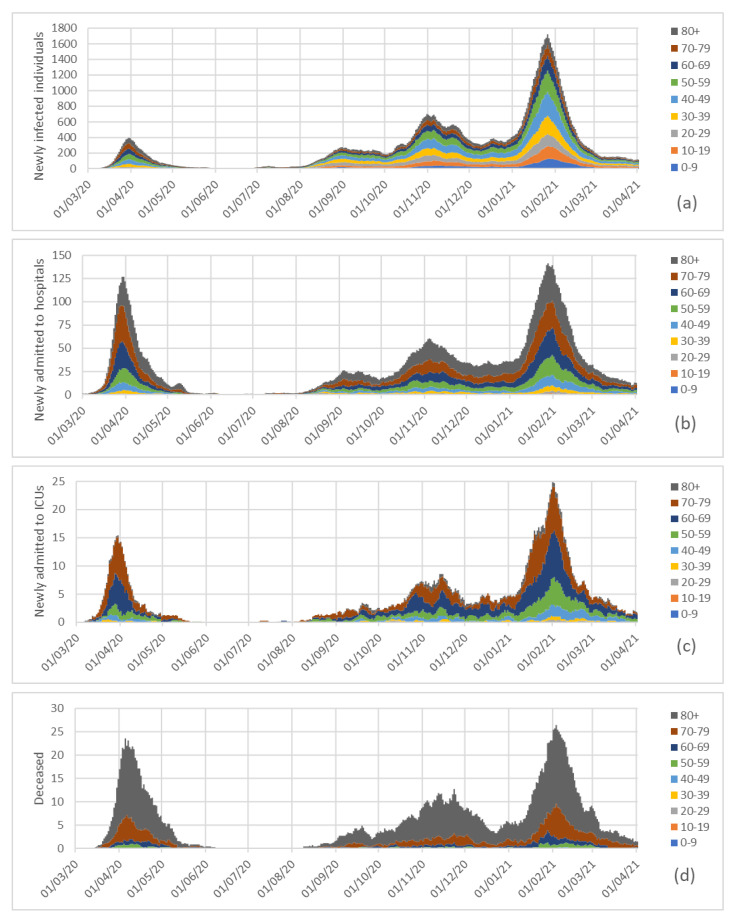
Newly infected individuals (**a**), admissions to hospitals (**b**), admissions to intensive care units (**c**) and deceased (**d**) in Galicia since the beginning of the pandemic, by considering age groups and means of the seven previous days.

**Figure 3:**
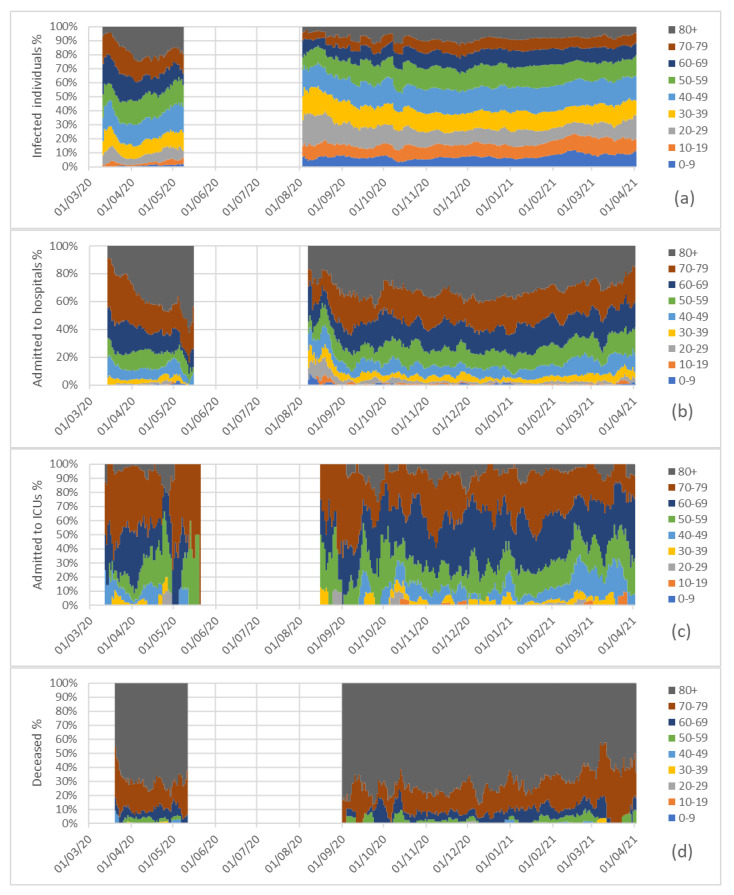
Percentages of newly infected (**a**), admissions to hospitals (**b**), admissions to intensive care units (**c**) and deceased (**d**) in Galicia since the beginning of the pandemic, by considering age groups and means of the seven previous days.

Figure 2 and Figure 3 include four stacked graphs with data and percentages for each variable, considering age groups and means of the seven previous days. In Figure 3, it is shown that some of the percentages between May and August 2020 were not reproduced because the data values were either zero (e.g., no infections or no deceased; see data in Figure 2) or very low, and therefore, we could not with statistically significance elaborate reliable percentages [21]. Avoiding using not statistically significant data prevents misinterpretation of results [22].

**Figure 4:**
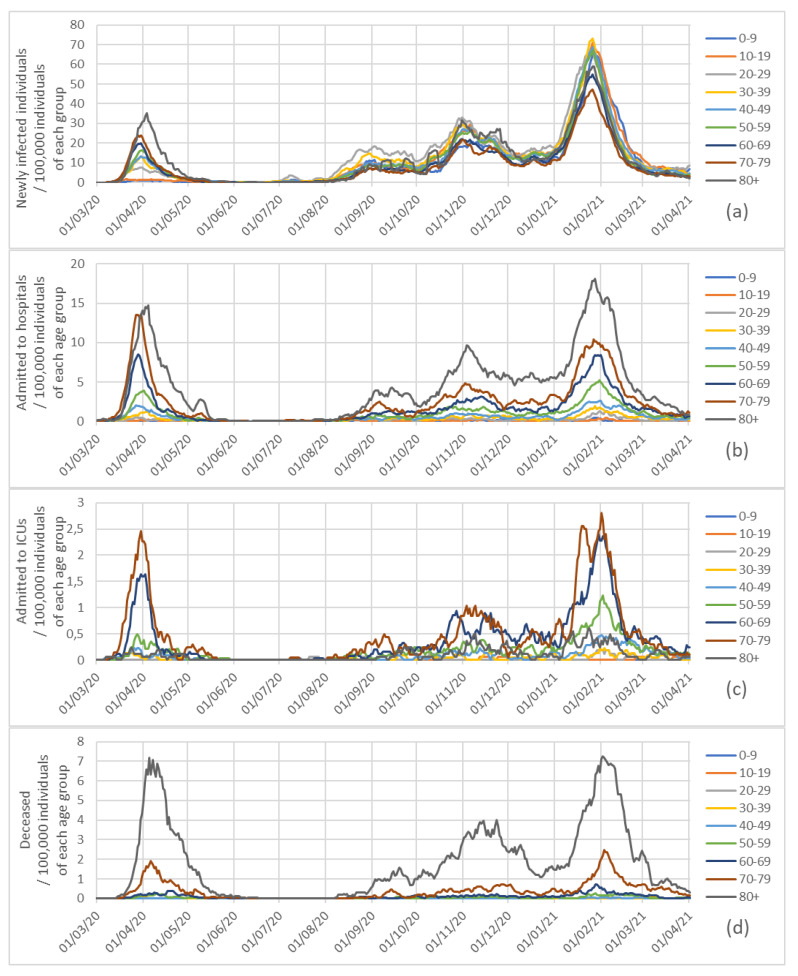
Newly infected (**a**), admissions to hospitals (**b**), admissions to intensive care units (**c**) and deceased (**d**) in Galicia for every 100,000 individuals of each group since the beginning of the pandemic, by considering age groups and means of the seven previous days.

**Figure 5:**
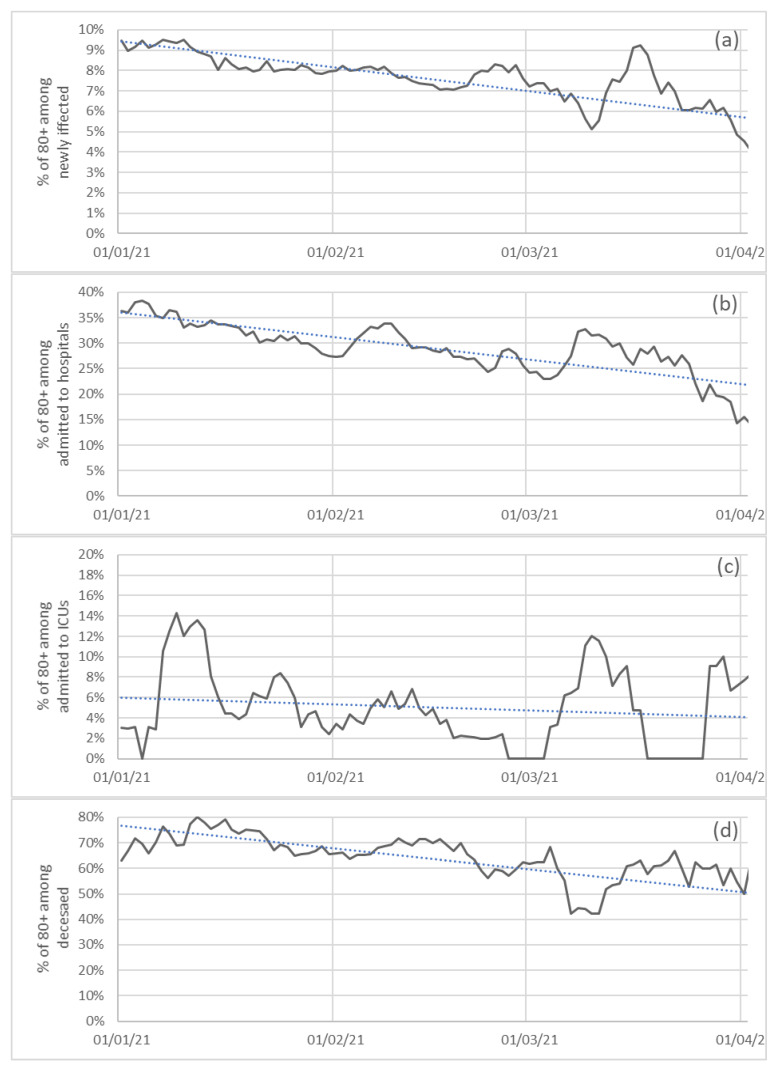
Percentages of 80+ among newly infected (**a**), admissions to hospitals (**b**), admissions to intensive care units (**c**) and deceased (**d**) in Galicia since 1st January 2021 for the age group 80+.

Figure 4 gives information about incidence/morbidity/mortality rate for every 100,000 individuals of each group, while also considering age groups and means of the seven previous days. The last four graphs are in Figure 5, showing the evolution of the 80+ age-group percentages among each variable since this group started vaccination.

Table 2, Table 3 and Table 4 include numerical data of the first wave, the full year of pandemic and since vaccinations started, respectively.

### 3.1. Newly Infected Individuals

Figure 2 represents the numbers of newly infected individuals from the beginning of the pandemic by age group for seven day periods. It is worth noticing that the maximum in February 2021 was about four times the maximum attained in April 2020. However, it must be taken into account that during the first wave, due to the lack a diagnosis strategy, the number of infected non-diagnosticated people would have been higher.

Moreover, Figure 3a shows the percentage of newly infected individuals of each age group during the full pandemic, and Figure 4a shows the newly infected individuals for every 100,000 individuals of each age group.

Globally, from the beginning of the pandemic until 31 July 2020, 9891 cases were detected in Galicia. Of these, only 121 corresponded to the 0–9 age group; 1753 corresponded to the 80-year-old age group. That is, 1% of cases corresponded to people under 10 years of age, representing 7.3% of the population, whereas 17.5% of cases were in people over 80 years of age, corresponding to only 8.69% of the population. Moreover, 6306 cases, i.e., almost 2/3 of the cases, were in people over 50, despite this population group being less than half of the population (47.5%). Data corresponding to the first wave have been included in Table 2.

By analyzing the complete period, we can observe that the number of infections in children below 9 years old experienced a significant increase when compared with the previous data, reaching 6.6% of the total, which may be related to the number of PCRs performed (even lower in the population it represents). However, the percentage of new infections in people over 80 is now 9.3%, possibly the result of vaccination (and deaths, age and previous pathologies complicated by COVID-19). Data corresponding to the full year have been included in Table 3.

In relation to vaccinations, we can observe that since 1 January 2021 there have been 54,245 new people infected, but the percentage of people over the age of 80 infected decreased considerably. We have the distribution included in Table 4.

As a final point for this subsection, in Figure 5 (a) we have included the evolution of the percentage of the group 80+ among the newly infected individuals, starting 1st January 2021. This is highly related to the beginning of the vaccination program which was initiated with the immunization of the older patients of sociosanitary residences and continued with people older than 80.

### 3.2. Admission at Hospitals

One of the major problems during the pandemic, mainly during the first wave, was to have resources to provide hospital care to all people who needed it.

During the first wave there were a total of 2842 hospitalizations, again concentrated in people over 50. It is also important to note that there were only six hospitalizations of children under 10. People over the age of 80 accounted for almost 31% of hospitalizations, and in addition, people over the age of 60 accounted for a total of 76.17% of hospitalizations during the first wave, while representing 32.44% of the total population. Data corresponding to the first wave have been included in Table 2.

In the entire pandemic period, there were a total of 12,555 hospitalizations with a higher percentage observed in the older age groups. Again, the group of people over 90 represented almost 32% of hospitalizations, and that of people over 60, 72.75% of them. This is summarized in Table 3, corresponding to the full year of the pandemic.

The number of admissions since the beginning of the pandemic and the percentages of admissions to hospitals by considering age groups are represented in Figure 2b and Figure 3b, and Figure 4b depicts the admissions to hospitals for every 100,000 individuals of each age group.

As in the previous case, in our opinion it is interesting to analyze only the period from 1 January 2021 onward, due to the effects of vaccination and the emergence of the new B.1.1.7 lineage (and deaths, age and previous pathologies complicated by COVID-19). In this time frame there were a total of 5100 hospitalizations, most of which (30.22%) were of people over 80. The percentage of people hospitalized over the age of 60 dropped from 71% in the second one. It is striking, compared to the first wave, that for the age group of children under 10 the percentage of hospitalizations rose from 0.21% to 0.35%. Table 4 reflects the data.

In Figure 5b we have included the evolution of the percentage of the 80+ group in terms of admission to hospitals, starting 1st January 2021. This was extremely influenced by the beginning of the vaccination program, which was initiated, as indicated before, with the immunization of the patients of sociosanitary residences and continued with people older than 80.

### 3.3. Admission at Intensive Care Units

One of the fundamental problems throughout the pandemic was being able to have sufficient resources in the health system to care for all sick people, not just COVID-19-infected people but all other patients. Preparing new ICU beds is not only a problem of physical and/or economical resources, but also of human resources, as ICU staff are highly specialized staff who need specific training and previous experience. Therefore, being able to predict the number of ICU beds has always been a priority, for which we use different methods. We will focus the data on new admissions to ICUs, not stay times.

During the first wave, a total of 315 people entered the ICU, according to the age groups shown in the Table 2. It can be observed that most of the people who had to be admitted to the ICU during the first wave (more than 42%) belonged to the age group between 70 and 79. Moreover, the sum of the percentages of 60–69 and 70–79 groups gives 72.7%, despite the fact that this group only represents 23.75% of the Galician population.

In the analysis of the entire pandemic period to date, we have a total of 1680 people who entered the ICU, of which the group between 60 and 79 represents the 64.35% of total income in the ICU, as summarized in Table 3.

In the period from 1 January 2021 to the present, the total number of cases was 803 (i.e., almost half, despite being a very short period); the distribution is included in Table 4.

Figure 2c and Figure 3c represent the number of admissions to ICUs in Galicia since the beginning of the pandemic, and the percentages of admissions to intensive care units by age group. Figure 4c represents the number of admissions to intensive care units per 100,000 individuals of each age group.

In Figure 5c we have included the evolution of the percentage of the group 80+ among new admissions to ICUs, starting 1 January 2021. In this case, the results should be interpreted with caution due to the small sample of 80+ patients admitted to ICUs (as deduced from Figure 5c and previous tables and data).

### 3.4. Deceased People

Finally, we analyzed the data on deceased people. Until June 2020 there were a total of 618 deaths, mainly of people over 80, who constituted almost 70.87% of deaths, despite being 8.69% of the Galician population. This is something that happens with other acute infectious diseases, although possibly not with the intensity of COVID-19. Moreover, no deaths of people under the age of 30 were recorded, with two cases in the 30–39 and 40–49 age groups, respectively. Data of the first wave has been included in Table 2.

If we now study the pandemic as a whole, we have the following distribution for the total of 2319 registered deceased individuals: 70.85% were in the group of people over 80 and over 90% in people over 70, as can be observed in Table 3.

Finally, in relation to the individuals who died after 1 January 2021, there have been 922 so far, which is a very high number in absolute terms if we consider that in the whole pandemic there were 2319 deaths. These data, included in Table 4, should be put in context in relation to the total infected people in that time period. The proportion of deaths over the age of 80 (67.03%) decreased compared to the first wave (70.87%) and the whole pandemic (70.85%).

In Figure 2d and Figure 3d we have represented the numbers of individuals who died (as a result of COVID-19) in Galicia since the beginning of the pandemic, and the percentages of deceased by age group; and Figure 4d presents the excess mortality for each age group per 100,000 individuals in each group, the rates in the 80+ group being similar during the maxima of the first and third waves (about seven daily deceased of every 100,000 individuals over 80).

In Figure 5d we have included the evolution of the percentage of the 80+ group among the deceased, starting 1 January 2021. This is extremely related to the beginning of the vaccination program, which was initiated with the immunization of the patients of sociosanitary residences and continued with people with older than 80.

## 4. Discussion

This was an extended descriptive study of the COVID-19 patients in Galicia, including newly infected individuals, admissions to hospitals, admissions to intensive care units and deceased individuals, by considering age groups. The four parameters have been analyzed in detail for the full period, starting 4 March 2020, and for the period starting in 1 January 2021. The latter is important due to two crucial issues: the spreading of the B.1.1.7 lineage and the starting of the vaccinating process.

Spain has 17 autonomous regions and two autonomous cities. As for the healthcare legislation, according to the Spanish Constitution, they can be (and in fact, they are) transferred to the autonomous regions, but three systems are in play, namely, Foreign Health, Bases and General Coordination of Health and Legislation on Pharmaceutical Products. Therefore, the data have been collected at the different autonomous regions and later sent to the central government, and finally published at the ISCIII. It is also important to note that, due to the political structure of Spain, the social distancing measures during the pandemic were established by the central government in the first period (from mid-March 2000 until June 2020), and later by the autonomous governments. Therefore, data from different autonomous regions may not be comparable. For this reason, and in order to maintain internal validity, we have focused our analysis in one of the autonomous regions, Galicia.

A so-called “weekend effect” has been observed in the registry of new infections: most Sunday data show local minima. Similar effects were not obvious in the other variables studied in this report, but cannot be ruled out for new admissions to hospital and deceased. To avoid the influence of the method of recording raw data, we recommend the use of 7-day means on the variables.

With the progression of the pandemic and the generalization of PCR tests among the population, the percentage of infected individuals of each age group tended to be close to its population percentage. Faced with this evidence, the record of infected persons under 30 years of age—and especially those under 20—during the first wave (4 March 2020 until mid-June 2020) appears out of place. This leads to think that in the months of March and April 2020 there was a large population of non-severe COVID cases who were not diagnosed due to a lack of PCR, and most relevantly, unimportant symptoms or none (about 2000 undetected vs. 9000 detected, 18%), who could have acted as vectors for the transmission of the disease. Percentages of newly infected individuals starting 1 January 2021 are quite similar to their proportions in the total population of Galicia. More precisely, the average of the absolute value of the differences between the percentage of newly infected individuals and the percentage of the corresponding age group is less than 1.3%, with a standard deviation of 0.8%.

Hospital admissions are merely testimonial in those individuals under 30 years of age, especially in those under 20. From that age range, the trend of a linear increase in these values is perceived as one advances in the age group, with a marked symmetry between the rise and fall of cases in each wave and age group. The exception occurs in the first wave and the 80+ group, where an anomalous asymmetry is documented in these data, which could be related to the rapid spread of the infection in nursing homes which led to a spike in resident transfers to hospitals. It is remarkable that admissions to hospital have the following variation for the 50+ group: As for the first wave, they represented about 88% of all admissions (85% in the full period); this value was reduced to 84.7% starting 1 January 2021. This group represents 47.55% of the total Galician population. On the other hand, as for the group of people younger than 30 years old, the percentage of admission at hospitals increased from 1.55% in the first wave, to 2.39% for the period starting 1 January 2021. This group represents 24.36% of the total Galicia population.

Regarding admissions to intensive care units, during the first wave the 50+ group represented more than 90% of admissions, which reduced to 85.68% in the period starting 1 January 2021. The percentage for the under 30 group increased from 1% to 1.25%. It has also been shown, with very conclusive figures and numbers, the triage system applied by hospitals: although the number of hospital admissions of the 80+ group has generally been higher than for the other groups, their presence in ICUs is hardly testimonial, being six times less than the 70+ and 60+ age groups, three times less than the 50+ age group and half of the 40+ age groups.

It is possible to observe that the percentages of deceased individuals, which was almost the same in the three periods for the 50+ groups: 99.35% in the first wave, 99.48% in the full pandemic and 99.36 in the period starting 1st January 2021. It is remarkable that there was only one dead in the full period for individuals in the under 30 group. It is necessary to draw attention to the devastating effect of the pandemic on those over 80, constituting 67% of the deceased, despite not reaching the 9% of the population of Galicia. Fortunately, the arrival of the vaccine to this age group brought an immediate improvement in this indicator in the first months of 2021.

We would like to emphasize some percentages for the 80+ group, which represents 8.69% of the total Galicia population. In the first wave they were 30.96% of the admissions to hospital, but only 3.81% of the admissions to intensive care units, and 70.87% of the deceased individuals. In the period starting January 1st, 2021, the percentage of admissions to hospital slightly decreased to 30.22% but the deceased up to 67.03%, probably due to the vaccination program.

As of 22 April 2021, the only age group with a significant percentage of vaccination was the 80+ group. For this age group, it has been possible to quantify the improvements of its indicators due to vaccination, by isolating it from the generalized improvements related to the partial overcoming of the third wave.

The impacts on morbidity and mortality of COVID-19 in the Galician community, in terms of infection, hospital admissions and mortality, are clearly associated with age—affecting in a very significant way in those over 80 years of age, rarely affecting the people under 30 (a single case) and seldom affecting in those under 50 (11 deaths, 0.5% of the 265 total). At the same time, the initiation of vaccination in older groups seems to have had an immediate impact on the evolution of the pandemic in terms of deaths.

With respect to the older population, it is the group over 80 years of age that, in proportion with its distribution in the general population, has the highest percentages of hospitalized and deceased. In fact, around 30% of the hospitalized population corresponds to this age range. This population is also the one with the highest risk of dying—70% of the deaths from COVID in the Autonomous Community were over 80 years old. These data do not differ from other epidemiological studies published to date [20,23].

Given that the impact of vaccines where it has demonstrated the most efficacy is avoiding or reducing the risk of mortality or admission [24,25], it seems very reasonable to adopt a vaccination strategy by age. In this sense, despite the fact that vaccination of those over 80 years old began on February 19, and ended on April 6, and therefore, at the closing date of the study, not the entire population over 80 years of age was with the completed vaccination schedule, there was evidence of a lower rate of hospitalization and less death from COVID. It is possible that, starting at the end of 2020, patients residing in social and healthcare homes—the profile of elderly people—helped achieve this impact.

The impact on children is residual. Thus, while the age group 0–9 represents 7.3% of the Galician population, the percentage of infected was 6.6%. In relation to hospital admission rates (0.35%), ICU (0%) and mortality (0%), all values were far lower, significantly, than the population averages. These data do not differ too much from other experiences already published [26,27,28,29] and reinforce the initial plan that vaccination is not a priority in these groups [30,31,32].

## 5. Conclusions

We have analyzed the evolution of the infection of the COVID-19 pandemic in Galicia (NW Spain) during the period from 1st March 2020 to 1st April 2021. We have considered four population compartments: new infected, hospital admissions, intensive care unit admissions and deceased. We have showed the relevance of the age group and this will be helpful in the future measures. It is reasonable to adopt a vaccination strategy by age. These findings add to the evidence of the determinants of COVID-19 infections of different risk factors.

## Figures and Tables

**Table 1:** Population of Galicia by age groups according to the “Instituto Galego de Estatística.” The percentage of each group has been also included.

Age Group	Total	Men	Women	%Total
0–9	196,986	101,798	95,189	7.30
10–19	229,357	118,485	110,872	8.50
20–29	231,018	118,104	112,914	8.56
30–39	315,034	158,033	157,002	11.67
40–49	442,773	220,633	222,140	16.41
50–59	407,874	199,372	208,502	15.11
60–69	350,336	167,931	182,404	12.98
70–79	290,725	131,827	158,898	10.77
80+	234,615	86,247	148,367	8.69
Total	2,698,718	1,302,430	1,396,288	

**Table 2:** Data corresponding to the first wave—4 March 2020 to mid-June 2020.

Age Group	0–9	10–19	20–29	30–39	40–49	50–59	60–69	70–79	80+
Newly infected (NI)	78	150	549	932	1428	1509	1456	1453	1734
%NI	0.84	1.61	5.91	10.03	15.37	16.25	15.67	15.64	18.67
Admission at hospital (AH)	6	10	28	84	217	332	527	758	880
%AH	0.21	0.35	0.99	2.96	7.64	11.68	18.54	26.67	30.96
Admission at ICU (AICU)	0	0	1	8	22	43	96	133	12
%AICU	0	0	0.32	2.54	6.98	13.65	30.48	42.22	3.81
Deceased	0	0	0	2	2	19	36	121	438
% Deceased	0	0	0	0.32	0.32	3.07	5.83	19.58	70.87
%Population	7.30	8.50	8.56	11.67	16.41	15.11	12.98	10.77	8.69

**Table 3:** Data corresponding to the full year—4 March 2020 to 1 April 2021.

Age Group	0–9	10–19	20–29	30–39	40–49	50–59	60–69	70–79	80+
Newly infected (NI)	7712	10,283	12,169	15,004	19,919	17,443	12,917	9848	10,836
%NI	6.64	8.85	10.48	12.92	17.15	15.02	11.12	8.48	9.33
Admission at hospital (AH)	55	69	203	477	1018	1599	2275	2853	4006
%AH	0.44	0.55	1.62	3.80	8.11	12.74	18.12	22.72	31.91
%Population	7.30	8.50	8.56	11.67	16.41	15.11	12.98	10.77	8.69
Admission at ICU (AICU)	0	5	13	47	151	290	534	547	93
%AICU	0	0.30	0.77	2.80	8.99	17.26	31.79	32.56	5.54
Deceased	0	0	1	4	7	68	150	446	1643
% Deceased	0	0	0.04	0.17	0.3	2.93	6.47	19.23	70.85
%Population	7.30	8.50	8.56	11.67	16.41	15.11	12.98	10.77	8.69

**Table 4:** Data corresponding to the period 1 January 2021 to 1 April 2021.

Age Group	0–9	10–19	20–29	30–39	40–49	50–59	60–69	70–79	80+
Newly infected (NI)	4307	5422	5303	6876	9667	8089	5953	4291	4388
%NI	7.93	9.99	9.76	12.67	17.81	14.89	10.97	7.90	8.09
Admission at hospital (AH)	18	25	79	206	453	695	979	1104	1541
%AH	0.35	0.49	1.55	4.04	8.88	13.63	19.2	21.65	30.22
Admission at ICU (AICU)	0	2	8	21	84	148	254	245	41
%AICU	0	0.25	1	2.62	10.46	18.43	31.63	30.51	5.11
Deceased	0	0	0	2	4	32	68	198	618
% Deceased	0	0	0	0.22	0.43	3.47	7.38	21.48	67.03
%Population	7.30	8.50	8.56	11.67	16.41	15.11	12.98	10.77	8.69

## Data Availability

The row data associated with this paper is available on-line in references [17,18,19]. It is also available on request from the corresponding author.
